# Identifying patient-centered communication barriers in palliative care

**DOI:** 10.1590/1980-220X-REEUSP-2025-0262en

**Published:** 2026-10-16

**Authors:** Betülay Kiliç, Ece Kafadar, Tuğba Yardimci Gürel

**Affiliations:** 1 University of Health Sciences, Gulhane Faculty of Nursing, Ankara, Turkey.; 2 Pursaklar State Hospital, Ankara Provincial Health Directorate, Ankara, Turkey.; 3 Başkent University, Faculty of Health Sciences, Department of Nursing, Ankara, Turkey.

**Keywords:** Palliative Care, Communication, Nursing, Communication Barriers, Nursing Research, Nursing Care, Cuidados Paliativos, Comunicação, Enfermagem, Barreiras de Comunicação, Pesquisa em Enfermagem, Cuidados de Enfermagem

## Abstract

**Objective:**

To identify patient-centered communication barriers among palliative care patients and to explore their relationship with sociodemographic and clinical characteristics.

**Method:**

This cross-sectional study was conducted with 124 patients hospitalized in the palliative care clinic of a state hospital. Data were collected using the Sociodemographic Data Form and the Patient-Centered Communication Barriers Scale. Mann-Whitney U test, Kruskal-Wallis test, and multiple linear regression analysis were used for statistical analysis.

**Results:**

Analysis showed that the presence of a caregiver was significantly associated with lower levels of insecurity (β = –0.542, p = 0.022, 95% CI [–1.001, –0.081]), explaining 7.6% of the variance in insecurity (Adjusted R^2^ = 0.076). However, the association between caregiver presence and perceived prejudice was not statistically significant (β = 0.202, p = 0.08).

**Conclusion:**

The findings indicate that palliative care patients experience notable communication barriers, particularly related to insecurity. Structured caregiver involvement may help reduce insecurity-related communication barriers and support more effective patient-centered communication in palliative care settings.

## RESUMO

**Objetivo:**

Identificar as barreiras à comunicação centrada no paciente entre pacientes em cuidados paliativos e explorar sua relação com características sociodemográficas e clínicas.

**Método:**

Estudo transversal realizado com 124 pacientes hospitalizados na clínica de cuidados paliativos de um hospital público. Os dados foram coletados por meio do Formulário de Dados Sociodemográficos e da Escala de Barreiras à Comunicação Centrada no Paciente. Para a análise estatística, foram utilizados os testes de Mann-Whitney U, Kruskal-Wallis e análise de regressão linear múltipla.

**Resultados:**

A análise mostrou que a presença de um cuidador esteve significativamente associada a menores níveis de insegurança (β = –0.542; p = 0.022; IC95% [–1.001; –0.081]), explicando 7,6% da variância da insegurança (R2 ajustado = 0.076). Entretanto, a associação entre a presença do cuidador e o preconceito percebido não foi estatisticamente significativa (β = 0.202; p = 0.08).

**Conclusão:**

Os achados indicam que pacientes em cuidados paliativos apresentam barreiras importantes à comunicação, especialmente relacionadas à insegurança. A participação estruturada do cuidador pode contribuir para reduzir as barreiras comunicacionais relacionadas à insegurança e favorecer uma comunicação mais efetiva centrada no paciente em contextos de cuidados paliativos.

## INTRODUCTION

Palliative care is a holistic healthcare service provided to improve the quality of life and ensure a more peaceful dying process for individuals with life-threatening illnesses. The primary goal of palliative care is not only to alleviate physical symptoms but also to address psychological, social, and spiritual needs^(1,2)^. The global increase in the aging population, rapid technological advancements, and improvements in the treatment of life-threatening diseases have extended life expectancy and, consequently, increased the need for palliative care^(3,4)^. Within this care process, effective communication between the patient and healthcare professionals is one of the most critical determinants of care quality. Effective communication enhances patient satisfaction, strengthens treatment adherence, and directly impacts the overall quality of healthcare services^(5)^. Communication is defined as a process of transferring knowledge, ideas, emotions, and experiences^(6)^. Many patients in palliative care experience various difficulties in establishing effective communication with healthcare providers. Common problems include difficulty in understanding or using the information provided, as well as limited recall of medical details. These issues are often linked to emotional and psychological stress experienced by patients. Additionally, individual factors such as advanced age, low educational level, and limited health literacy can exacerbate these challenges^(7,8)^. Yet, patients often consider good communication with healthcare providers as one of the most vital elements in palliative care; insufficient communication may lead to decreased patient satisfaction, disrupted continuity of care, reduced patient safety, and interruptions in shared decision-making processes^(9)^. Factors such as patients’ emotional states, uncertain prognoses, cultural differences, time constraints, and lack of communication training among healthcare professionals further complicate this process^(10)^. Communication in palliative care is not limited to the patient alone; family members and caregivers also become active participants in the process. Especially in end-of-life situations, families or those involved in the patient’s care often communicate directly with healthcare professionals and are engaged in decision-making^(11,12)^. This dynamic makes communication more complex, requiring the management of different information needs and emotional reactions. The concept of patient-centered care is grounded in respecting individuals’ values, preferences, and needs. This approach supports patients’ active participation in treatment and requires healthcare professionals to communicate empathetically and respectfully. In palliative care, adopting a patient-centered approach goes beyond ensuring physical comfort it aims to bring meaning to both life and the dying process^(1)^. However, various barriers are often encountered in patient-centered communication. In a qualitative study conducted in the Netherlands, it was reported that general practitioners faced major challenges in managing patients’ emotional responses, meeting family demands, and managing time effectively^(5)^. Similarly, significant personal uncertainties-such as not knowing which doctor would be responsible, wanting to focus on living rather than dying, and being unsure of preferred care-were identified as major barriers to end-of-life discussions among patients with advanced Chronic Obstructive Pulmonary Disease^(10)^.

The competency of nurses in providing patient-centered care plays a direct role in the quality of palliative care delivered. In addition to patient-related factors, barriers to effective communication in palliative care often stem from provider-level and systemic challenges. A qualitative study conducted in Massachusetts, USA, it is revealed that healthcare and community-based service providers face significant difficulties in engaging patients in conversations about advance care planning (ACP) and palliative care^(13)^. These challenges included a lack of provider knowledge and preparedness, discomfort with serious illness discussions, and structural inconsistencies such as regional differences in ACP policies and limited institutional support. Furthermore, misconceptions about palliative care and cultural and language barriers were identified as common obstacles across multiple settings. These findings underscore the complexity of communication in palliative care, highlighting the need for provider training, policy-level improvements, and culturally sensitive communication strategies to ensure that such conversations occur early, effectively, and with patient-centered intent^(13)^. Despite extensive literature addressing communication challenges in palliative care, most studies focus on healthcare professionals’ experiences, general communication difficulties, or family perspectives rather than barriers perceived directly by patients. Moreover, little is known about how specific patient-related factors such as caregiver presence, sociodemographic characteristics, and clinical conditions shape patient-centered communication barriers. Quantitative studies using validated tools to assess these relationships remain limited. As a result, there is a gap in understanding how palliative care patients themselves experience and interpret communication barriers within the healthcare system, and which individual or contextual factors contribute to these challenges. Addressing this gap is essential for developing targeted communication strategies that align with patients’ needs and expectations. In this context, identifying patient-centered communication barriers that hinder effective interaction in palliative care services is of critical importance for both theoretical knowledge development and clinical application. This study investigated the level of patient-centered communication barriers among palliative care patients and examined the relationship between these barriers and patients’ sociodemographic and clinical characteristics. The findings are expected to contribute to the development of strategies aimed at strengthening communication between patients and healthcare providers in palliative care. Moreover, they may serve as a guide for creating communication-focused training programs, particularly for nurses, who are integral to this process.

## METHODS

### Study Design and Sample

This study was designed as a cross-sectional study. It was conducted with patients hospitalized in the Palliative Care Clinic of a State Hospital in Ankara, Turkey, between December 2022 and December 2024. The reporting of the study adhered to the Strengthening the Reporting of Observational Studies in Epidemiology (STROBE) guidelines for cross-sectional research^(14)^. Participants were included if they: (1) were 18 years of age or older, (2) were hospitalized in the Palliative Care Clinic during the data collection period, (3) had adequate cognitive capacity to participate, including the absence of acute confusional states (delirium), and (4) had sufficient reading, language comprehension, and verbal communication abilities to understand and complete the study questionnaires. Participants were excluded if they: (1) had a documented diagnosis of dementia or intellectual disability that could impair comprehension of study instruments, or (2) had any clinical condition that prevented reliable communication as judged by the clinical team. All eligible individuals who met the criteria and agreed to participate provided written informed consent. The study was completed with a total of 124 participants. To evaluate the adequacy of the sample size, a post hoc power analysis was conducted using G*Power (version 3.1). The analysis showed that the sample provided a statistical power of 0.95 at a significance level of α = 0.05 and a 95% confidence interval. These results indicate that the sample size was sufficient to detect meaningful differences and that the study had a low likelihood of a Type II error.

### Data Collection

Data were collected by the researchers through face-to-face interviews using the Sociodemographic Data Form and the Patient-Centered Communication Barriers Scale (PCCBS). Prior to data collection, all participants were informed about the purpose and procedures of the study and were asked to sign an informed consent form. The timing of the interviews was scheduled by considering patients’ treatment and care routines, and interviews were conducted during periods when patients were most likely to be physically and mentally available. The data collection instruments were filled out by the researcher due to the patients responses. Researchers remained present in the clinic throughout the process to address any questions or provide clarification when needed. On average, the data collection process for each participant took approximately 15 to 20 minutes to complete.

### Sociodemographic Data Form

The sociodemographic data form filled out by the patients participating in the study consisted of seven questions and included questions on the year of birth, gender, marital status, income level, educational status, presence of caregiver, presence of comorbidities, and symptom experience.

### Patient-Centered Communication Barriers Scale (PCCBS)

The Patient-Centered Communication Barriers Scale (PCCBS) was developed by Şahin et al.^(15)^ to identify communication barriers experienced by patients within hospital settings. The scale comprises 28 items and seven subdimensions confirmed through exploratory and confirmatory factor analyses: need for attention, security staff, fear and anxiety, concern and need for being informed, waiting time, prejudice, and lack of trust toward healthcare personnel. The Need for Attention domain reflects patients’ expectations for empathy, understanding, and emotional support from healthcare personnel. Security Staff captures perceptions of how security personnel contribute to or hinder a safe and communicative care environment. Fear and Anxiety assesses emotional states such as worry about illness, prognosis, or medical procedures that may interfere with communication. Concern and Need for Being Informed measures patients’ curiosity and need for clear, accessible information about their health status and treatment steps. Waiting Time captures discomfort and communication breakdowns triggered by delays in examinations, procedures, or care processes. The Prejudice domain consists of four negatively worded items that evaluate patients’ perceptions of unfairness, favoritism, neglect, or discriminatory attitudes from healthcare staff. Because these items express negative experiences, they are reverse-scored so that their direction aligns with the other domains. After reverse scoring, higher scores uniformly indicate greater communication barriers across all subdimensions. Finally, Lack of Trust assesses reduced confidence in the competence, decisions, and professional integrity of healthcare personnel, which can directly impede effective communication.

The scale consists of 6 items on need for attention, 4 on security staff, 4 on fear and anxiety, 4 on concern and need for information, 3 on waiting time, 4 on prejudice, and 3 on lack of trust. All items are rated on a 5-point Likert scale ranging from “Strongly disagree” to “Strongly agree.” In the scoring system developed by Şahin et al.^(15)^, items in the need for attention, security staff, fear and anxiety, concern and need for information, waiting time, and lack of trust domains are scored from 1 to 5, whereas the four prejudice items being negatively worded are reverse-scored to ensure that higher values consistently reflect greater perceived communication barriers. Subdimension scores are calculated by dividing the sum of item scores within each domain by the number of items, and the overall scale score is obtained by dividing the sum of all item scores by 28. The maximum obtainable score is 140, with higher averages indicating more substantial patient-perceived barriers to communication.

### Data Analysis

Statistical analyses were performed using IBM SPSS Statistics for Windows, Version 24.0 (IBM Corp.; Armonk, NY, USA, Released 2016). The normality of numerical variables was assessed using the Shapiro-Wilk test. Descriptive analyses (frequency, percentage, mean, and standard deviation) were used to describe the characteristics of the patients and the scores they obtained from the scales. Cronbach’s alpha reliability coefficient was calculated to determine the internal consistency of the scale scores. Mann-Whitney U and Kruskal-Wallis tests were employed to examine differences between independent variables and total scores on the measurement tool. Multiple linear regression analysis was performed to examine factors associated with patient-centered communication barriers. The dependent variables were the insecurity and prejudice sub-dimensions. Independent variables included caregiver presence and variables that showed statistical significance in univariate analyses (p < 0.05). These variables were entered into the regression model using the enter method. Model fit was evaluated using R^2^ and adjusted R^2^ values, and statistical significance was set at p < 0.05.

### Ethical Aspects

The study received approval from the Ethics Committee of the Training and Research Hospital (Decision No: 2022/90). Participants were provided with detailed verbal and written information regarding the study’s objectives and procedures, and written informed consent was obtained from all participants prior to data collection. The study was conducted in accordance with the ethical principles outlined in the Declaration of Helsinki. Given that participants were highly vulnerable, additional precautions were taken to ensure their protection and well-being: all data were anonymized and personal identifiers removed to maintain confidentiality; questionnaires were administered in a manner considerate of patients’ comfort and clinical condition, with breaks provided as needed; participants were informed of their right to withdraw from the study at any time without affecting their medical care; and in case of any distress during data collection, participants were offered immediate support by the healthcare team. These measures ensured the study adhered to ethical standards while safeguarding the rights and well-being of this vulnerable population.

## RESULTS

The mean age of participants was 73.33 ± 16.33 years, with 53.2% of participants being female. A majority of the participants (96.2%) reported the presence of symptoms. Table 1 presents the sociodemographic and clinical characteristics of the participants.

**Table 1:** Sociodemographic and clinical characteristics of the participants (n = 124) – Ankara, Turkey, 2024.

Characteristics		
Age (years)	(Mean ± SD) 73.33 ± 16.33	Min–Max (19–97)
Gender	n	%
Male	58	46.8
Female	66	53.2
Educational Status		
Primary school	96	77.5
High school	21	16.9
University and over	7	6.5
Marital status		
Married	63	50.8
Single	61	49.2
Presence of Caregiver		
Yes	112	90.3
No	12	9.7
Income and expense status		
Income more than expense	47	37.9
Equal income and expense	51	41.1
Income less than expense	26	21.0
Presence of Symptoms		
No	4	3.8
Yes	120	96.2

SD = Standard deviation.

Table 2 presents the mean scores of the patient-centered communication barriers scale. The highest mean values were observed in the subdimensions of “need for attention”, and “desire for curiosity and information,” whereas the lowest mean was observed in the “prejudice” subdimension.

**Table 2:** The mean scores of the patient-centered communication barrier scale (n = 124) – Ankara, Turkey, 2024.

	n	Mean	SD	Min.	Max.
Total	124	3.70	0.41	2.46	4.93
Need for attention	124	4.40	0.59	1.83	5.0
Security stuff	124	3.68	0.76	1.0	5.0
Fear and anxiety	124	3.62	0.93	1.0	5.0
Concern and need for being informed	124	4.0	0.63	1.25	5.0
Waiting times	124	3.60	0.97	1.0	5.0
Prejudice	124	2.32	1.09	1.0	5.0
Lack of trust towards healthcare personnel	124	3.95	0.72	1.0	5.0

SD = Standard deviation.

In Table 3, scale scores were compared based on sociodemographic and clinical characteristics using Mann-Whitney U and Kruskal-Wallis tests. A statistically significant difference was observed in relation to the presence of a caregiver (p = 0.034; p = 0.041). Insecurity was higher among participants with a caregiver, whereas prejudice was more pronounced among those without caregiver support.

**Table 3:** Comparison of scale scores with sociodemographic and clinical characteristics (n = 124) – Ankara, Turkey, 2024.

Variable	Scale total score	Need for attention	Curiosity and need for knowledge	Waiting time	Insecurity	Security personnel	Fear and anxiety	Prejudice
	Median (Q1–Q3)	Median (Q1–Q3)	Median (Q1–Q3)	Median (Q1–Q3)	Median (Q1–Q3)	Median (Q1–Q3)	Median (Q1–Q3)	Median (Q1–Q3)
Gender								
Female Male Test*/p	3.75 (3.45–3.96) 3.66 (3.49–3.85) –1.123/0.262	4.83 (4.0–5.0) 4.0 (4.0–5.0) –1.418/0.156	4.0 (3.75–4.56) 4.0 (3.75–4.0) –1.106/0.269	3.6 (2.66–4.0) 4.0 (3.25–4.66) –1.602/0.109	4.0 (3.91–4.66) 4.0 (3.33–4.0) –1.880/0.060	3.87 (3.25–4.25) 3.5 (3.25–4.06) –1.047/0.295	4.0 (3.18–4.0) 4.0 (3.43–4.25) –1.116/0.264	2.25 (1.75–3.06) 2.0 (1.25–2.75) –1.908/0.052
Marital Status								
Married Single Test*/p	3.71 (3.42–3.96) 3.67 (3.48–3.89) –0.355/0.7233	4.5 (4.0–5.0) 4.5 (4.9–5.0) –0.473/0.636	4.0 (3.75–4.5) 4.0 (3.75–4.0) –1.010/0.312	3.66 (3.0–4.0) 4.0 (3.0–4.3) –0.319/0.750	4.0 (1.66–2.33) 4.0 (1.83–2.33) –0.493/0.622	3.75 (3.66–4.33) 3.5 (3.66–4.16) –0.708/0.479	4.0 (3.0–4.25) 4.0 (3.25–4.0) –0.564/0.573	2.25 (1.75–3.25) 2.0 (1.0–2.62) –1.347/0.178
Education Status								
Primary education High School University and above Test**/p	3.66 (3.42–3.89) 3.82 (3.55–3.96) 3.78 (3.46–4.35) 1.839/0.399	4.5 (4.0–5.0) 4.5 (4.0–5.0) 5.0 (3.83–5.0) 0.435/0.805	4.0 (3.75–4.18) 4.0 (3.75–4.37) 4.0 (3.75–4.75) 0.157 /0.925	3.66 (2.66–4.0) 4.0 (3.16–4.33) 4.0 (3.66–4.66) 1.508/0.470	4.0 (3.66–4.33) 4.0 (3.5–4.16) 4.0 (3.66–4.0) 0.652/0.722	3.5 (3.25–4.0) 4.0 (3.25–4.25) 3.25 (3.0–5.0) 0.360/ 0.835	4.0 (3.06–4.0) 4.0 (3.5–4.12) 4.0 (3.75–4.75) 2.221/0.329	2.0 (1.06–3.0) 2.25 (1.37–3.12) 2.0 (2.0–3.0) 0.623/0.733
Income Level								
Income less than expenditure Income equal to expenditure Income more than expenditure Test**/p	3.71 (3.39–3.96) 3.67 (3.46–3.89) 3.67 (3.52–3.86) 0.095/0.954	4.5 (4.0–5.0) 4.5 (4.0–5.0) 4.58 (4.0–5.0) 1.668/0.434	4.0 (3.75–4.0) 4.0 (3.75–4.5) 4.0 (3.75–4.0) 0.438/0.803	4.0 (2.66–4.0) 3.66 (3.0–4.0) 3.83 (3.33–4.66) 0.675/0.714	4.0 (4.0–4.33) 4.0 (3.66–4.33) 4.0 (3.33–4.0) 1.282/0.527	3.5 (3.25–4.0) 4.0 (3.25–4.24) 3.5 (3.5–4.0) 2.935/0.230	4.0 (3.5–4.0) 3.75 (3.0–4.0) 4.0 (3.68–4.0) 1.979/0.372	2.25 (1.75–3.25) 2.0 (1.0–3.0) 2.12 (1.0–2.75) 0.860/0.651
Caregiver								
Yes No Test*/p	3.67 (3.46–3.89) 3.75 (3.60–3.95) –0.524/0.601	4.5 (4.0–5.0) 4.0 (4.0–4.95) –0.529/0.597	4.0 (3.75–4.25) 3.87 (3.75–4.0) –1.124/0.261	3.66 (3.0–4.0) 4.0 (3.25–4.66) –1.651/0.099	4.0 (3.66–4.33) 3.83 (3.33–4.0) –2.119/0.034	3.75 (3.25–4.25) 3.25 (3.06–4.0) –1.275/0.202	4.0 (3.06–4.0) 4.0 (3.75–4.0) –0.403/0.687	2.0 (1.0–3.0) 2.37 (2.0–3.12) –2.006/ 0.041
Symptom Experience Status								
Yes No Test*/p	3.67 (3.46–3.89) 3.75 (2.75–4.04) –0.028/0.977	4.5 (4.0–5.0) 4.66 (3.25–4.95) –0.029/0.977	4.0 (4.0–5.0) 4.25 (3.25–4.95) –1.022/0.982	4.0 (3.0–4.0) 3.16 (1.91–4.66) –0.680/0.496	4.0 (3.66–4.0) 4.5 (3.75–5.0) –1.135/0.256	3.75 (3.25–4.18) 3.87 (3.31–4.81) –0.794/0.427	4.0 (3.25–4.0) 3.0 (1.62–4.0) –1.160/0.246	2.12 (1.75–3.0) 1.37 (1.0–2.68) –1.287/0.198

Data represented either as median (Q1–Q3); Note: p-value: Mann-Whitney U* and Kruskal-Wallis** tests.

Table 4 presents the results of the multiple linear regression analyses examining factors associated with patient-centered communication barriers. The regression model predicting insecurity was statistically significant (F = 1.777, p = 0.022) and explained 7.6% of the variance in insecurity (Adjusted R^2^ = 0.076). The presence of a caregiver was significantly associated with lower levels of insecurity (B = –0.542, β = –0.222, p = 0.022, 95% CI [–1.001, –0.081]). In contrast, the regression model predicting prejudice was not statistically significant (F = 2.740, p = 0.08), and the presence of a caregiver was not significantly associated with perceived prejudice.

**Table 4:** Presence of a caregiver and its association with insecurity and prejudice – Ankara, Turkey, 2024.

Independent variable	Dependent variable	Unstandardized coefficients			Standardized coefficients			95% CI	
		B	SE	Adjusted R^2^	ß	t	p	Lower	Upper
Presence of a caregiver	Prejudice	0.202	0.115	0.116	0.166	1.752	0.080	–0.02	0.43
Presence of a caregiver	Insecurity	–0.542	0.233	0.076	–0.222	–2.326	**0.022**	–1.001	–0.081

SE = Standard error; CI = Confidence interval.

## DISCUSSION

This study aimed to identify patient-centered communication barriers among individuals receiving palliative care and to examine their relationship with sociodemographic and clinical characteristics. The findings revealed that patients experience challenges related to the need for information, the expectation of attention, and distrust toward healthcare staff. The presence of a caregiver was significantly associated with lower levels of insecurity in communication with healthcare professionals, suggesting a supportive role of caregivers in reducing communication-related concerns. In the multiple linear regression analysis, which included variables significant in univariate analyses, caregiver presence was the only variable significantly associated with insecurity. However, caregiver presence was not significantly associated with perceived prejudice, indicating that this dimension may be influenced by factors beyond caregiver involvement. Effective communication not only enhances patient satisfaction but also improves treatment adherence and the overall quality of healthcare services.

In our study, the mean scores of the subdimensions of “need for attention” and “desire for curiosity and information” were higher than other subdimensions, indicating that patients value both technical care and human connection, as well as clear, explanatory information from healthcare professionals. No statistical comparisons were performed to determine whether these differences are significant. Recognition of patients’ communication demands by nurses can lead to a shift toward more patient-centered and palliative-oriented care goals^(16)^. Structured communication guides can promote earlier and more meaningful end-of-life care discussions among patients, caregivers, and healthcare professionals^(17)^. Major barriers to effective communication in palliative care include the healthcare provider’s inability to initiate communication, time constraints, lack of an appropriate setting, and patients’ difficulty in understanding medical language^(5)^. Difficulties in effective communication about palliative care may also arise due to cultural differences, insufficient training, and complex interactions with family caregivers^(13)^. Disharmony in end-of-life communication is frequently experienced by nurses and is often attributed to a lack of knowledge, misunderstanding of terminology, and mismatched expectations between patients and families. Terms such as “hospice” or “palliative care” are often perceived as synonymous with giving up or dying, highlighting the need for careful word choice and empathetic communication^(7)^.

In palliative care, trust-based relationships form the foundation of effective communication. Building such trust between healthcare professionals and family members facilitates not only the transmission of information but also enhances families’ understanding of the patient’s worsening condition, promotes their participation in end-of-life decision-making, and ensures that care preferences are respected. A trustful environment encourages mutual understanding and openness, which contributes to dignified and patient-centered decision-making processes. Therefore, communication strategies in palliative care should be designed not only to convey information but also to provide emotional support and foster trust^(18,19)^.

The subdimension “prejudice” had the lowest mean score among the subdimensions. Respect and social connectedness are central components in older adults’ definitions of “good care” during the end-of-life period^(20)^. This observation aligns with our study and underscores the importance of fostering respectful and dignity-preserving communication styles in patientcentered care.

One notable finding of this study is the relationship between caregiver presence and communication barriers. Regression analysis showed that caregiver presence was significantly associated with lower levels of insecurity, whereas its association with perceived prejudice was not statistically significant. The reduction in insecurity suggests that caregiver involvement may help alleviate patients’ concerns about being understood, receiving adequate attention, and obtaining clear and reliable information from healthcare professionals. This finding is consistent with studies indicating that family involvement in the care process may have both facilitating and complicating effects on communication^(20)^. Caregivers may act as supportive intermediaries by assisting patients in articulating their needs, clarifying information exchanged during clinical encounters, and providing emotional reassurance. In palliative care settings, where patients often experience heightened emotional vulnerability, such support may contribute to a greater sense of security in communication with healthcare personnel. However, caregiver presence was not significantly related to perceived prejudice, suggesting that this dimension of communication barriers may be shaped by broader contextual factors beyond interpersonal support alone. As noted in the scale development study, perceptions of prejudice are influenced by factors such as institutional trust, perceived quality of care, and patients’ previous healthcare experiences^(15)^. Although caregiver presence significantly predicted insecurity, the proportion of variance explained by the regression model was relatively modest (Adjusted R^2^ = 7.6%), indicating that communication barriers in palliative care are multifactorial and complex. Factors such as fluctuating emotional states, illness severity, symptom burden, and organizational characteristics of care settings may interact with caregiver involvement in shaping patients’ communication experiences. Therefore, caregiver presence should be interpreted as one contributing factor within a broader psychosocial and organizational framework rather than as a sole determinant of patient-centered communication barriers.

No significant differences were found between demographic variables (e.g., gender, marital status, education, income) and communication barriers, suggesting that communication experiences are influenced more by the structure of the healthcare system and the communication skills of providers than by individual characteristics. Institutional care culture plays a key role in determining the quality of communication^(16)^. Similarly, institutions where nurses have high levels of patient-centered communication competence tend to show greater patient satisfaction and higher quality of care^(1)^.

One of the most valuable aspects of this study is its use of self-reported data obtained directly from palliative care patients. These individuals face numerous physical and psychosocial challenges during the most vulnerable and fragile period of their lives. Understanding patients’ communication experiences based on their own perceptions plays a crucial role not only in clinical decision-making but also in shaping the overall philosophy of care. In this context, patient-reported evaluations serve as a powerful resource for informing and improving healthcare services^(9)^. Furthermore, enhancing patient engagement contributes to more effective information exchange and leads to improved care quality overall^(17)^. These findings are consistent with our results and highlight the substantial influence of communication experiences on both clinical practice and the broader approach to care.

Four distinct nurse approaches to patient-centered communication in end-of-life care have been identified: empathetic listener, family-oriented supporter, resource-utilizing facilitator, and time-investing holistic caregiver. These classifications illustrate how individual nurse communication styles can shape patient experiences. The empathetic listener approach, in particular, aligns with the need for feeling understood, which was frequently emphasized by patients in our study^(8)^.

One of the most essential elements of effective communication in palliative care is ensuring that individuals coping with intractable suffering feel understood. A qualitative study demonstrated the vital role of supportive communication and empathy in this process. Participants reported that they not only developed a better understanding of others’ suffering but also confronted their own feelings of inadequacy, thereby becoming more resilient and effective communicators. Tolerating moments of silence, in particular, allowed patients to express their feelings and thoughts more freely, opening the door to a sense of inner peace for both the patient and the caregiver^(21)^. These findings emphasize that communication in palliative care must be grounded not only in technical knowledge-sharing but also in emotional resilience and mutual understanding, which aligns closely with the present study’s results.

This study has several limitations. The study was conducted at a single center, which limits the generalizability of the findings to other institutions, regions, or healthcare systems. Furthermore, the cross-sectional design prevents the establishment of causal relationships between variables and allows only the identification of associations. The use of self-reported data may also have introduced bias related to participants’ emotional or cognitive conditions, including recall bias. Future studies should include multicenter and more diverse samples, use longitudinal designs, and incorporate qualitative methods to provide a deeper understanding of communication experiences in palliative care. Despite these limitations, the study has several strengths. It used the Patient-Centered Communication Barriers Scale to assess communication barriers and obtained self-reported data directly from palliative care patients. This approach provides first-hand insight into patients’ communication experiences during a particularly vulnerable period and contributes a patient-centered perspective that is often underrepresented in palliative care research.

These findings have important implications for clinical practice. Healthcare professionals working in palliative care settings should be supported with structured communication training programs that emphasize empathy, clarity, and active patient engagement. Addressing patients’ unmet needs for attention and information may help reduce communication barriers and improve the overall care experience. In addition, caregivers can play a supportive role in facilitating communication by helping patients express their concerns and better understand the information provided. However, it is important to ensure that caregiver involvement does not overshadow the patient’s own voice in the communication process. Strengthening patient participation in information-sharing and decision-making processes may further enhance patient-centered communication in palliative care settings.

## CONCLUSION

This study identified key patient-centered communication barriers among individuals receiving palliative care, particularly unmet needs for attention and information. The findings indicate that the presence of a caregiver is associated with lower levels of insecurity in communication with healthcare professionals, suggesting a potential supportive role of caregivers in alleviating patients’ communication-related concerns. However, caregiver presence was not significantly associated with perceived prejudice, indicating that this dimension of communication barriers may be influenced by factors beyond caregiver involvement alone.

## Data Availability

The entire dataset supporting the results of this study is available upon request to the corresponding author.
