# Supplementary material for: Identifying patient-centered communication barriers in palliative care
Source: Rev Esc Enferm USP. 2026 Oct 16;60:e20250262. doi: 10.1590/1980-220X-REEUSP-2025-0262en (PMC13682498; doi:10.1590/1980-220X-REEUSP-2025-0262en)
Supplement: Translation

# Translation: Identificación de las barreras en la comunicación centrada en el paciente en los cuidados paliativos

**DOI:** 10.1590/1980-220X-REEUSP-2025-0262es

**Authors:** Betülay Kiliç, Ece Kafadar, Tuğba Yardimci Gürel

**Affiliations:** 1 University of Health Sciences, Gulhane Faculty of Nursing, Ankara, Turkey.; 2 Pursaklar State Hospital, Ankara Provincial Health Directorate, Ankara, Turkey.; 3 Başkent University, Faculty of Health Sciences, Department of Nursing, Ankara, Turkey.

**Keywords:** Cuidados Paliativos, Comunicación, Enfermería, Barreras de Comunicación, Investigación en Enfermería, Atención de Enfermería

## Abstract

**Objetivo:**

Identificar las barreras de comunicación centradas en el paciente entre los pacientes de cuidados paliativos y explorar su relación con las características sociodemográficas y clínicas.

**Método:**

Este estudio transversal se llevó a cabo con 124 pacientes hospitalizados en la clínica de cuidados paliativos de un hospital estatal. Los datos se recolectaron mediante el Formulario de Datos Sociodemográficos y la Escala de Barreras de Comunicación Centrada en el Paciente. Para el análisis estadístico se utilizaron la prueba U de Mann-Whitney, la prueba de Kruskal-Wallis y el análisis de regresión lineal múltiple.

**Resultados:**

El análisis mostró que la presencia de un cuidador se asoció significativamente con niveles más bajos de inseguridad (β = –0,542, p = 0,022, IC del 95% [–1,001, –0,081]), lo que explica el 7,6% de la varianza en la inseguridad (R^2^ ajustado = 0,076). Sin embargo, la asociación entre la presencia de un cuidador y el prejuicio percibido no fue estadísticamente significativa (β = 0,202, p = 0,08).

**Conclusión:**

Los hallazgos indican que los pacientes en cuidados paliativos experimentan barreras de comunicación notables, particularmente relacionadas con la inseguridad. La participación estructurada de los cuidadores puede ayudar a reducir las barreras de comunicación relacionadas con la inseguridad y promover una comunicación más efectiva centrada en el paciente en entornos de cuidados paliativos.

## INTRODUCCIÓN

Los cuidados paliativos son un servicio de salud integral que se brinda para mejorar la calidad de vida y garantizar un proceso de muerte más tranquilo para las personas con enfermedades que ponen en riesgo la vida. El objetivo principal de los cuidados paliativos no es solo aliviar los síntomas físicos, sino también atender las necesidades psicológicas, sociales y espirituales^(1,2)^. El aumento global del envejecimiento de la población, los rápidos avances tecnológicos y las mejoras en el tratamiento de enfermedades que ponen en riesgo la vida han prolongado la esperanza de vida y, en consecuencia, han incrementado la necesidad de cuidados paliativos^(3,4)^.

Dentro de este proceso de atención, la comunicación efectiva entre el paciente y los profesionales de la salud es uno de los determinantes más críticos de la calidad de la atención. La comunicación efectiva mejora la satisfacción del paciente, refuerza la adherencia al tratamiento y tiene un impacto directo en la calidad general de los servicios de salud^(5)^. La comunicación se define como un proceso de transferencia de conocimientos, ideas, emociones y experiencias^(6)^. Muchos pacientes en cuidados paliativos experimentan diversas dificultades para establecer una comunicación efectiva con los proveedores de salud. Entre los problemas comunes se incluyen la dificultad para comprender o utilizar la información proporcionada, así como la memoria limitada de los detalles médicos. Estos problemas suelen estar relacionados con el estrés emocional y psicológico que experimentan los pacientes. Además, factores individuales como la edad avanzada, el bajo nivel educativo y la alfabetización en salud limitada pueden agravar estos desafíos^(7,8)^.

Sin embargo, los pacientes suelen considerar que una buena comunicación con los profesionales de la atención de salud es uno de los elementos más importantes en los cuidados paliativos; una comunicación insuficiente puede conducir a una disminución de la satisfacción del paciente, a la interrupción de la continuidad de la atención, a una menor seguridad del paciente y a interrupciones en los procesos de toma de decisiones compartida^(9)^. Factores como el estado emocional de los pacientes, los pronósticos inciertos, las diferencias culturales, las limitaciones de tiempo y la falta de capacitación en comunicación entre los profesionales de la salud complican aún más este proceso^(10)^. La comunicación en los cuidados paliativos no se limita únicamente al paciente; los familiares y los cuidadores también se convierten en participantes activos en el proceso. Especialmente en situaciones de fin de vida, las familias o las personas involucradas en el cuidado del paciente suelen comunicarse directamente con los profesionales de la salud y participan en la toma de decisiones^(11,12)^. Esta dinámica hace que la comunicación sea más compleja, ya que requiere manejar diferentes necesidades de información y reacciones emocionales.

El concepto de atención centrada en el paciente se basa en el respeto a los valores, las preferencias y las necesidades de las personas. Este enfoque fomenta la participación activa de los pacientes en el tratamiento y exige que los profesionales de la salud se comuniquen con empatía y respeto. En los cuidados paliativos, adoptar un enfoque centrado en el paciente va más allá de garantizar el bienestar físico: busca dar sentido tanto a la vida como al proceso de morir^(1)^. Sin embargo, a menudo se encuentran diversas barreras en la comunicación centrada en el paciente. En un estudio cualitativo realizado en los Países Bajos, se informó que los médicos de familia enfrentaban grandes desafíos para manejar las respuestas emocionales de los pacientes, satisfacer las demandas de la familia y administrar el tiempo de manera eficaz^(5)^. De manera similar, se identificaron importantes incertidumbres personales —como no saber qué médico estaría a cargo, querer enfocarse en vivir en lugar de en morir y no estar seguro de la atención preferida— como obstáculos importantes para las conversaciones sobre el final de la vida entre pacientes con enfermedad pulmonar obstructiva crónica en etapa avanzada^(10)^.

La competencia de las enfermeras para brindar atención centrada en el paciente influye directamente en la calidad de los cuidados paliativos que se prestan. Además de los factores relacionados con el paciente, las barreras para una comunicación efectiva en los cuidados paliativos a menudo se derivan de desafíos a nivel de los proveedores y de la sistema. Un estudio cualitativo realizado en Massachusetts, EE. UU., reveló que los proveedores de servicios de salud y comunitarios enfrentan dificultades significativas para involucrar a los pacientes en conversaciones sobre la planificación anticipada de la atención (PAA) y los cuidados paliativos^(13)^.

Estos desafíos incluían la falta de conocimiento y preparación de los proveedores, la incomodidad al hablar sobre enfermedades graves e inconsistencias estructurales, tales como diferencias regionales en las políticas de PAA y un apoyo institucional limitado. Además, se identificaron conceptos erróneos sobre los cuidados paliativos y barreras culturales y lingüísticas como obstáculos comunes en múltiples entornos. Estos hallazgos subrayan la complejidad de la comunicación en los cuidados paliativos, lo que pone de relieve la necesidad de capacitación para los proveedores, mejoras a nivel de políticas y estrategias de comunicación que tengan en cuenta las diferencias culturales, a fin de garantizar que dichas conversaciones se produzcan de manera temprana, eficaz y con un enfoque centrado en el paciente^(13)^.

A pesar de la extensa literatura que aborda los desafíos de comunicación en los cuidados paliativos, la mayoría de los estudios se centran en las experiencias de los profesionales de la salud, las dificultades generales de comunicación o las perspectivas de las familias, en lugar de en las barreras percibidas directamente por los pacientes. Además, se sabe poco sobre cómo factores específicos relacionados con los pacientes —como la presencia de cuidadores, las características sociodemográficas y las condiciones clínicas— influyen en las barreras de comunicación centradas en el paciente. Los estudios cuantitativos que utilizan herramientas validadas para evaluar estas relaciones siguen siendo limitados.

Como resultado, existe una brecha en la comprensión de cómo los propios pacientes de cuidados paliativos experimentan e interpretan las barreras de comunicación dentro del sistema de salud, y qué factores individuales o contextuales contribuyen a estos desafíos. Abordar esta brecha es esencial para desarrollar estrategias de comunicación específicas que se ajusten a las necesidades y expectativas de los pacientes. En este contexto, identificar las barreras de comunicación centradas en el paciente que dificultan la interacción efectiva en los servicios de cuidados paliativos es de vital importancia tanto para el desarrollo del conocimiento teórico como para la aplicación clínica.

Este estudio investigó el nivel de barreras de comunicación centradas en el paciente entre los pacientes de cuidados paliativos y examinó la relación entre estas barreras y las características sociodemográficas y clínicas de los pacientes. Se espera que los hallazgos contribuyan al desarrollo de estrategias destinadas a fortalecer la comunicación entre los pacientes y los proveedores de atención médica en los cuidados paliativos. Además, pueden servir como guía para crear programas de capacitación enfocados en la comunicación, particularmente para el personal de enfermería, que es parte integral de este proceso.

## MÉTODOS

### Diseño del Estudio y Muestra

Este estudio se diseñó como un estudio transversal. Se llevó a cabo con pacientes hospitalizados en la Clínica de Cuidados Paliativos de un hospital estatal en Ankara, Turquía, entre diciembre de 2022 y diciembre de 2024. El informe del estudio se ajustó a las directrices de Fortalecimiento de la Presentación de Estudios Observacionales en Epidemiología (STROBE) para la investigación transversal^(14)^. Se incluyó a los participantes si: (1) tenían 18 años de edad o más, (2) estaban hospitalizados en la Clínica de Cuidados Paliativos durante el período de recolección de datos, (3) contaban con la capacidad cognitiva adecuada para participar, lo que incluía la ausencia de estados de confusión agudos (delirio), y (4) tenían habilidades suficientes de lectura, comprensión del lenguaje y comunicación verbal para entender y completar los cuestionarios del estudio.

Se excluyó a los participantes si: (1) tenían un diagnóstico documentado de demencia o discapacidad intelectual que pudiera afectar la comprensión de los instrumentos del estudio, o (2) presentaban alguna afección clínica que impidiera una comunicación confiable, según el criterio del equipo clínico. Todas las personas elegibles que cumplieron con los criterios y aceptaron participar otorgaron su consentimiento informado por escrito. El estudio se completó con un total de 124 participantes. Para evaluar la adecuación del tamaño de la muestra, se realizó un análisis de potencia post hoc utilizando G*Power (versión 3.1). El análisis mostró que la muestra proporcionaba una potencia estadística de 0,95 con un nivel de significación de α = 0,05 y un intervalo de confianza del 95%. Estos resultados indican que el tamaño de la muestra era suficiente para detectar diferencias significativas y que el estudio tenía una baja probabilidad de cometer un error de tipo II.

### Recolección de Datos

Los investigadores recolectaron los datos mediante entrevistas presenciales utilizando el Formulario de Datos Sociodemográficos y la Escala de Barreras de Comunicación Centrada en el Paciente (PCCBS). Antes de la recolección de datos, se informó a todos los participantes sobre el propósito y los procedimientos del estudio y se les pidió que firmaran un formulario de consentimiento informado. El horario de las entrevistas se programó teniendo en cuenta las rutinas de tratamiento y atención de los pacientes, y las entrevistas se llevaron a cabo durante los períodos en los que era más probable que los pacientes estuvieran disponibles física y mentalmente. Los instrumentos de recolección de datos fueron completados por el investigador en base a las respuestas de los pacientes. Los investigadores permanecieron presentes en la clínica durante todo el proceso para responder cualquier pregunta o brindar aclaraciones cuando fuera necesario. En promedio, el proceso de recolección de datos para cada participante tomó aproximadamente de 15 a 20 minutos.

### Formulario de Datos Sociodemográficos

El formulario de datos sociodemográficos que completaron los pacientes que participaron en el estudio constaba de siete preguntas e incluía preguntas sobre el año de nacimiento, el género, el estado civil, el nivel de ingresos, el nivel educativo, la presencia de un cuidador, la presencia de comorbilidades y los síntomas experimentados.

### Escala de Barreras de Comunicación Centrada en el Paciente (Patient-Centered Communication Barriers Scale - PCCBS)

La Escala de Barreras de Comunicación Centrada en el Paciente (PCCBS) fue desarrollada por Şahin et al.^(15)^ para identificar las barreras de comunicación que experimentan los pacientes en entornos hospitalarios. La escala comprende 28 ítems y siete subdimensiones confirmadas mediante análisis factoriales exploratorios y confirmatorios: necesidad de atención, personal de seguridad, miedo y ansiedad, preocupación y necesidad de estar informado, tiempo de espera, prejuicios y falta de confianza hacia el personal de salud. El dominio “Necesidad de atención” refleja las expectativas de los pacientes respecto a la empatía, la comprensión y el apoyo emocional por parte del personal de atención de salud. El dominio “Personal de seguridad” capta las percepciones sobre cómo el personal de seguridad contribuye a crear o dificulta un ambiente de atención seguro y propicio para la comunicación. El dominio “Miedo y ansiedad” evalúa estados emocionales como la preocupación por la enfermedad, el pronóstico o los procedimientos médicos que pueden interferir en la comunicación. El dominio “Preocupación y necesidad de estar informado” mide la curiosidad de los pacientes y su necesidad de información clara y accesible sobre su estado de salud y los pasos del tratamiento. El “Tiempo de espera” recoge la incomodidad y las fallas en la comunicación provocadas por retrasos en los exámenes, procedimientos o procesos de atención. El dominio “Prejuicio” consta de cuatro ítems redactados de manera negativa que evalúan las percepciones de los pacientes sobre la injusticia, el favoritismo, el descuido o las actitudes discriminatorias por parte del personal de salud. Dado que estos ítems expresan experiencias negativas, se les aplica una puntuación inversa para que su dirección se alinee con la de los demás dominios. Tras la puntuación inversa, las puntuaciones más altas indican de manera uniforme mayores barreras de comunicación en todas las subdimensiones. Por último, la “Falta de confianza” evalúa la disminución de la confianza en la competencia, las decisiones y la integridad profesional del personal de salud, lo cual puede obstaculizar directamente una comunicación efectiva.

La escala consta de 6 ítems sobre la necesidad de atención, 4 sobre el personal de seguridad, 4 sobre el miedo y la ansiedad, 4 sobre la preocupación y la necesidad de información, 3 sobre el tiempo de espera, 4 sobre los prejuicios y 3 sobre la falta de confianza. Todos los ítems se califican en una escala de Likert de 5 puntos que va desde “Totalmente en desacuerdo” hasta “Totalmente de acuerdo”.

En el sistema de puntuación desarrollado por Şahin et al.^(15)^, los ítems de los dominios “necesidad de atención”, “personal de seguridad”, “miedo y ansiedad”, “preocupación y necesidad de información”, “tiempo de espera” y “falta de confianza” se califican del 1 al 5, mientras que los cuatro ítems de “prejuicio”, que están redactados de forma negativa, se califican a la inversa para garantizar que los valores más altos reflejen de manera consistente mayores barreras de comunicación percibidas. Las puntuaciones de las subdimensiones se calculan dividiendo la suma de las puntuaciones de los ítems dentro de cada dominio por el número de ítems, y la puntuación global de la escala se obtiene dividiendo la suma de todas las puntuaciones de los ítems entre 28. La puntuación máxima que se puede obtener es 140, y los promedios más altos indican barreras de comunicación más sustanciales percibidas por los pacientes.

### Análisis de Datos

Los análisis estadísticos se realizaron utilizando IBM SPSS Statistics para Windows, versión 24.0 (IBM Corp.; Armonk, NY, EE. UU., lanzado en 2016). La normalidad de las variables numéricas se evaluó mediante la prueba de Shapiro-Wilk. Se utilizaron análisis descriptivos (frecuencia, porcentaje, media y desviación estándar) para describir las características de los pacientes y las puntuaciones que obtuvieron en las escalas. Se calculó el coeficiente de confiabilidad alfa de Cronbach para determinar la consistencia interna de las puntuaciones de la escala. Se emplearon las pruebas U de Mann-Whitney y de Kruskal-Wallis para examinar las diferencias entre las variables independientes y las puntuaciones totales en la herramienta de medición. Se realizó un análisis de regresión lineal múltiple para examinar los factores asociados con las barreras de comunicación centrada en el paciente. Las variables dependientes fueron las subdimensiones de inseguridad y prejuicio. Las variables independientes incluyeron la presencia de un cuidador y las variables que mostraron significación estadística en los análisis univariados (p < 0,05). Estas variables se introdujeron en el modelo de regresión utilizando el método “enter”. El ajuste del modelo se evaluó mediante los valores de R^2^ y R^2^ ajustado, y la significación estadística se estableció en p < 0,05.

### Aspectos Éticos

El estudio recibió la aprobación del Comité de Ética del Hospital de Capacitación e Investigación (Decisión N.º: 2022/90). Se proporcionó a los participantes información detallada, tanto verbal como por escrito, sobre los objetivos y procedimientos del estudio, y se obtuvo el consentimiento informado por escrito de todos los participantes antes de la recolección de datos. El estudio se llevó a cabo de conformidad con los principios éticos establecidos en la Declaración de Helsinki. Dado que los participantes eran altamente vulnerables, se tomaron precauciones adicionales para garantizar su protección y bienestar: todos los datos se anonimizaron y se eliminaron los identificadores personales para mantener la confidencialidad; los cuestionarios se administraron de manera que se tomara en cuenta la comodidad y el estado clínico de los pacientes, con descansos según fuera necesario; se informó a los participantes de su derecho a retirarse del estudio en cualquier momento sin que ello afectara su atención médica; y, en caso de cualquier malestar durante la recolección de datos, el equipo de atención médica ofreció apoyo inmediato a los participantes. Estas medidas aseguraron que el estudio se ajustara a los estándares éticos, al tiempo que salvaguardaban los derechos y el bienestar de esta población vulnerable.

## RESULTADOS

La edad promedio de los participantes fue de 73,33 ± 16,33 años, y el 53,2% de los participantes eran mujeres. La mayoría de los participantes (96,2%) reportó la presencia de síntomas. La Tabla 1 presenta las características sociodemográficas y clínicas de los participantes.

**Tabla 1:** Características sociodemográficas y clínicas de los participantes (n = 124) – Ankara, Turquía, 2024.

Características		
Edad (años)	(Media ± DE) 73,33 ± 16,33	Mín.–Máx. (19–97)
Sexo	n	%
Hombres	58	46,8
Mujeres	66	53,2
Nivel educativo		
Educación primaria	96	77,5
Secundaria	21	16,9
Universidad y estudios superiores	7	6,5
Estado civil		
Casado	63	50,8
Soltero	61	49,2
Presencia de un cuidador		
Sí	112	90,3
No	12	9,7
Situación de ingresos y gastos		
Los ingresos superan a los gastos	47	37,9
Ingresos e gastos iguales	51	41,1
Ingresos inferiores a los gastos	26	21,0
Presencia de síntomas		
No	4	3,8
Sí	120	96,2

DE = Desviación estándar.

La Tabla 2 presenta las puntuaciones medias de la escala de barreras de comunicación centrada en el paciente. Los valores medios más altos se observaron en las subdimensiones «necesidad de atención» y «deseo de curiosidad e información», mientras que la media más baja se observó en la subdimensión «prejuicio».

**Tabla 2:** Puntuaciones medias de la escala de barreras de comunicación centrada en el paciente (n = 124) – Ankara, Turquía, 2024.

	n	Media	DE	Mín.	Máx.
Total	124	3,70	0,41	2,46	4,93
Necesidad de atención	124	4,40	0,59	1,83	5,0
Cuestiones de seguridad	124	3,68	0,76	1,0	5,0
Miedo y ansiedad	124	3,62	0,93	1,0	5,0
Preocupación y necesidad de estar informado	124	4,0	0,63	1,25	5,0
Tiempos de espera	124	3,60	0,97	1,0	5,0
Prejuicio	124	2,32	1,09	1,0	5,0
Falta de confianza hacia el personal sanitario	124	3,95	0,72	1,0	5,0

DE = Desviación estándar.

En la Tabla 3, se compararon las puntuaciones de la escala en función de las características sociodemográficas y clínicas mediante las pruebas U de Mann-Whitney y de Kruskal-Wallis. Se observó una diferencia estadísticamente significativa en relación con la presencia de un cuidador (p = 0,034; p = 0,041). La inseguridad fue mayor entre los participantes con un cuidador, mientras que los prejuicios fueron más pronunciados entre aquellos que no contaban con el apoyo de un cuidador.

**Tabla 3:** Comparación de las puntuaciones de la escala con las características sociodemográficas y clínicas (n = 124) – Ankara, Turquía, 2024.

Variable	Puntuación total de la escala	Necesidad de atención	Curiosidad y necesidad de conocimiento	Tiempo de espera	Inseguridad	Personal de seguridad	Miedo y ansiedad	Prejuicio
	Mediana (Q1–Q3)	Mediana (Q1–Q3)	Mediana (Q1–Q3)	Mediana (Q1–Q3)	Mediana (Q1–Q3)	Mediana (Q1–Q3)	Mediana (Q1–Q3)	Mediana (Q1–Q3)
Género								
Mujer Hombre Prueba*/p	3,75 (3,45–3,96) 3,66 (3,49–3,85) –1,123/0,262	4,83 (4,0–5,0) 4,0 (4,0–5,0) –1,418/0,156	4,0 (3,75–4,56) 4,0 (3,75–4,0) –1,106/0,269	3,6 (2,66–4,0) 4,0 (3,25–4,66) –1,602/0,109	4,0 (3,91–4,66) 4,0 (3,33–4,0) –1,880/0,060	3,87 (3,25–4,25) 3,5 (3,25–4,06) –1,047/0,295	4,0 (3,18–4,0) 4,0 (3,43–4,25) –1,116/0,264	2,25 (1,75–3,06) 2,0 (1,25–2,75) –1,908/0,052
Estado civil								
Casado Soltero Prueba*/p	3,71 (3,42–3,96) 3,67 (3,48–3,89) –0,355/0,7233	4,5 (4,0–5,0) 4,5 (4,9–5,0) –0,473/0,636	4,0 (3,75–4,5) 4,0 (3,75–4,0) –1,010/0,312	3,66 (3,0–4,0) 4,0 (3,0–4,3) –0,319/0,750	4,0 (1,66–2,33) 4,0 (1,83–2,33) –0,493/0,622	3,75 (3,66–4,33) 3,5 (3,66–4,16) –0,708/0,479	4,0 (3,0–4,25) 4,0 (3,25–4,0) –0,564/0,573	2,25 (1,75–3,25) 2,0 (1,0–2,62) –1,347/0,178
Nivel educativo								
Educación primaria Educación secundaria Universitaria y superior Prueba**/p	3,66 (3,42–3,89) 3,82 (3,55–3,96) 3,78 (3,46–4,35) 1,839/0,399	4,5 (4,0–5,0) 4,5 (4,0–5,0) 5,0 (3,83–5,0) 0,435/0,805	4,0 (3,75–4,18) 4,0 (3,75–4,37) 4,0 (3,75–4,75) 0,157/0,925	3,66 (2,66–4,0) 4,0 (3,16–4,33) 4,0 (3,66–4,66) 1,508/0,470	4,0 (3,66–4,33) 4,0 (3,5–4,16) 4,0 (3,66–4,0) 0,652/0,722	3,5 (3,25–4,0) 4,0 (3,25–4,25) 3,25 (3,0–5,0) 0,360/0,835	4,0 (3,06–4,0) 4,0 (3,5–4,12) 4,0 (3,75–4,75) 2,221/0,329	2,0 (1,06–3,0) 2,25 (1,37–3,12) 2,0 (2,0–3,0) 0,623/0,733
Nivel de ingresos								
Ingresos inferiores a los gastos Ingresos iguales a los gastos Ingresos superiores a los gastos Prueba**/p	3,71 (3,39–3,96) 3,67 (3,46–3,89) 3,67 (3,52–3,86) 0,095/0,954	4,5 (4,0–5,0) 4,5 (4,0–5,0) 4,58 (4,0–5,0) 1,668/0,434	4,0 (3,75–4,0) 4,0 (3,75–4,5) 4,0 (3,75–4,0) 0,438/0,803	4,0 (2,66–4,0) 3,66 (3,0–4,0) 3,83 (3,33–4,66) 0,675/0,714	4,0 (4,0–4,33) 4,0 (3,66–4,33) 4,0 (3,33–4,0) 1,282/0,527	3,5 (3,25–4,0) 4,0 (3,25–4,24) 3,5 (3,5–4,0) 2,935/0,230	4,0 (3,5–4,0) 3,75 (3,0–4,0) 4,0 (3,68–4,0) 1,979/0,372	2,25 (1,75–3,25) 2,0 (1,0–3,0) 2,12 (1,0–2,75) 0,860/0,651
Cuidador								
Sí No Prueba*/p	3,67 (3,46–3,89) 3,75 (3,60–3,95) –0,524/0,601	4,5 (4,0–5,0) 4,0 (4,0–4,95) –0,529/0,597	4,0 (3,75–4,25) 3,87 (3,75–4,0) –1,124/0,261	3,66 (3,0–4,0) 4,0 (3,25–4,66) –1,651/0,099	4,0 (3,66–4,33) 3,83 (3,33–4,0) –2,119/ 0,034	3,75 (3,25–4,25) 3,25 (3,06–4,0) –1,275/0,202	4,0 (3,06–4,0) 4,0 (3,75–4,0) –0,403/0,687	2,0 (1,0–3,0) 2,37 (2,0–3,12) –2,006/0,041
Estado de los síntomas								
Sí No Prueba*/p	3,67 (3,46–3,89) 3,75 (2,75–4,04) –0,028/0,977	4,5 (4,0–5,0) 4,66 (3,25–4,95) –0,029/0,977	4,0 (4,0–5,0) 4,25 (3,25–4,95) –1,022/0,982	4,0 (3,0–4,0) 3,16 (1,91–4,66) –0,680/0,496	4,0 (3,66–4,0) 4,5 (3,75–5,0) –1,135/0,256	3,75 (3,25–4,18) 3,87 (3,31–4,81) –0,794/0,427	4,0 (3,25–4,0) 3,0 (1,62–4,0) –1,160/0,246	2,12 (1,75–3,0) 1,37 (1,0–2,68) –1,287/0,198

Datos representados como mediana (Q1–Q3); Nota: valor p: pruebas de Mann-Whitney U* y de Kruskal-Wallis**.

La Tabla 4 presenta los resultados de los análisis de regresión lineal múltiple que examinan los factores asociados a las barreras de la comunicación centrada en el paciente. El modelo de regresión que predice la inseguridad fue estadísticamente significativo (F = 1,777; p = 0,022) y explicó el 7,6% de la varianza en la inseguridad (R^2^ ajustado = 0,076). La presencia de un cuidador se asoció de forma significativa con niveles más bajos de inseguridad (B = –0,542, β = –0,222, p = 0,022, IC del 95% [–1,001; –0,081]). Por el contrario, el modelo de regresión que predecía los prejuicios no fue estadísticamente significativo (F = 2,740, p = 0,08), y la presencia de un cuidador no se asoció de forma significativa con los prejuicios percibidos.

**Tabla 4:** Presencia de un cuidador y su asociación con la inseguridad y los prejuicios – Ankara, Turquía, 2024.

Variable independiente	Variable dependiente	Coeficientes no estandarizados			Coeficientes estandarizados			IC del 95 %	
		B	SE	R R^2^	ß	t	p	Inferior	Superior
Presencia de un cuidador	Prejuicio	0,202	0,115	0,116	0,166	1,752	0,080	–0,02	0,43
Presencia de un cuidador	Inseguridad	–0,542	0,233	0,076	–0,222	–2,326	**0,022**	–1,001	–0,081

SE = error estándar; IC = intervalo de confianza.

## DISCUSIÓN

El objetivo de este estudio fue identificar las barreras de comunicación centradas en el paciente entre las personas que reciben cuidados paliativos y examinar su relación con las características sociodemográficas y clínicas. Los resultados revelaron que los pacientes enfrentan desafíos relacionados con la necesidad de información, la expectativa de atención y la desconfianza hacia el personal de salud. La presencia de un cuidador se asoció significativamente con menores niveles de inseguridad en la comunicación con los profesionales de la salud, lo que sugiere que los cuidadores desempeñan un papel de apoyo en la reducción de las preocupaciones relacionadas con la comunicación. En el análisis de regresión lineal múltiple, que incluyó las variables significativas en los análisis univariados, la presencia de un cuidador fue la única variable asociada de manera significativa con la inseguridad. Sin embargo, la presencia de un cuidador no se asoció de manera significativa con el prejuicio percibido, lo que indica que esta dimensión puede verse influida por factores más allá de la participación del cuidador. La comunicación efectiva no solo aumenta la satisfacción del paciente, sino que también mejora el cumplimiento del tratamiento y la calidad general de los servicios de salud.

En nuestro estudio, las puntuaciones promedio de las subdimensiones «necesidad de atención» y «deseo de curiosidad e información» fueron más altas que las de otras subdimensiones, lo que indica que los pacientes valoran tanto la atención técnica como la conexión humana, así como la información clara y explicativa por parte de los profesionales de la salud. No se realizaron comparaciones estadísticas para determinar si estas diferencias son significativas. El reconocimiento de las necesidades de comunicación de los pacientes por parte del personal de enfermería puede conducir a un cambio hacia objetivos de atención más centrados en el paciente y orientados a los cuidados paliativos^(16)^. Las guías de comunicación estructuradas pueden promover conversaciones más tempranas y significativas sobre los cuidados al final de la vida entre los pacientes, los cuidadores y los profesionales de la salud^(17)^. Entre los principales obstáculos para una comunicación efectiva en los cuidados paliativos se encuentran la incapacidad del proveedor de atención médica para iniciar la comunicación, las limitaciones de tiempo, la falta de un entorno adecuado y la dificultad de los pacientes para comprender el lenguaje médico^(5)^. Las dificultades para una comunicación efectiva sobre los cuidados paliativos también pueden surgir debido a diferencias culturales, capacitación insuficiente e interacciones complejas con los cuidadores familiares^(13)^. Las enfermeras suelen experimentar desarmonía en la comunicación sobre el final de vida, lo cual a menudo se atribuye a la falta de conocimiento, a la mala interpretación de la terminología y a expectativas desalineadas entre los pacientes y sus familias. Términos como «hospicio» o «cuidados paliativos» suelen percibirse como sinónimos de rendirse o morir, lo que resalta la necesidad de elegir cuidadosamente las palabras y de mantener una comunicación empática^(7)^.

En los cuidados paliativos, las relaciones basadas en la confianza constituyen la base de una comunicación eficaz. Fomentar esa confianza entre los profesionales de la salud y los familiares no solo facilita la transmisión de información, sino que también mejora la comprensión de las familias respecto al empeoramiento del estado del paciente, promueve su participación en la toma de decisiones sobre el final de la vida y garantiza que se respeten las preferencias de atención. Un entorno de confianza fomenta el entendimiento mutuo y la franqueza, lo que contribuye a procesos de toma de decisiones dignos y centrados en el paciente. Por lo tanto, las estrategias de comunicación en los cuidados paliativos deben diseñarse no solo para transmitir información, sino también para brindar apoyo emocional y fomentar la confianza^(18,19)^.

La subdimensión «prejuicio» obtuvo la puntuación media más baja entre las subdimensiones. El respeto y la conexión social son componentes centrales en las definiciones que dan las personas mayores de lo que es una «buena atención» durante el período del final de vida^(20)^. Esta observación concuerda con nuestro estudio y subraya la importancia de fomentar estilos de comunicación respetuosos y que preserven la dignidad en la atención centrada en el paciente.

Un hallazgo notable de este estudio es la relación entre la presencia del cuidador y las barreras de comunicación. El análisis de regresión mostró que la presencia del cuidador se asoció significativamente con niveles más bajos de inseguridad, mientras que su asociación con el prejuicio percibido no fue estadísticamente significativa. La reducción de la inseguridad sugiere que la participación de los cuidadores puede ayudar a aliviar las preocupaciones de los pacientes respecto a ser comprendidos, recibir la atención adecuada y obtener información clara y confiable de los profesionales de la salud. Este hallazgo concuerda con estudios que indican que la participación de la familia en el proceso de atención puede tener efectos tanto facilitadores como complicadores en la comunicación^(20)^. Los cuidadores pueden actuar como intermediarios de apoyo al ayudar a los pacientes a expresar sus necesidades, aclarar la información intercambiada durante las consultas clínicas y brindar tranquilidad emocional. En entornos de cuidados paliativos, donde los pacientes suelen experimentar una mayor vulnerabilidad emocional, dicho apoyo puede contribuir a una mayor sensación de seguridad en la comunicación con el personal de salud. Sin embargo, la presencia de los cuidadores no se relacionó significativamente con el prejuicio percibido, lo que sugiere que esta dimensión de las barreras de comunicación puede estar determinada por factores contextuales más amplios que van más allá del apoyo interpersonal por sí solo. Como se señaló en el estudio de desarrollo de la escala, las percepciones de prejuicio están influenciadas por factores tales como la confianza en la institución, la calidad percibida de la atención y las experiencias previas de los pacientes con el sistema de salud^(15)^. Aunque la presencia del cuidador predijo de manera significativa la inseguridad, la proporción de varianza explicada por el modelo de regresión fue relativamente modesta (R^2^ ajustado = 7,6%), lo que indica que las barreras de comunicación en los cuidados paliativos son multifactoriales y complejas. Factores como los estados emocionales fluctuantes, la gravedad de la enfermedad, la carga de los síntomas y las características organizativas de los entornos de atención pueden interactuar con la participación del cuidador para dar forma a las experiencias de comunicación de los pacientes. Por lo tanto, la presencia del cuidador debe interpretarse como un factor contribuyente dentro de un marco psicosocial y organizativo más amplio, en lugar de como un determinante único de las barreras de comunicación centradas en el paciente.

No se encontraron diferencias significativas entre las variables demográficas (p. ej., género, estado civil, nivel educativo, ingresos) y las barreras de comunicación, lo que sugiere que las experiencias de comunicación están más influenciadas por la estructura del sistema de salud y las habilidades de comunicación de los proveedores que por las características individuales. La cultura de atención institucional desempeña un papel clave en la determinación de la calidad de la comunicación^(16)^. De manera similar, las instituciones en las que el personal de enfermería cuenta con altos niveles de competencia en comunicación centrada en el paciente tienden a mostrar una mayor satisfacción de los pacientes y una mejor calidad de la atención^(1)^.

Uno de los aspectos más valiosos de este estudio es el uso de datos autoinformados obtenidos directamente de pacientes en cuidados paliativos. Estas personas enfrentan numerosos desafíos físicos y psicosociales durante el período más vulnerable y frágil de sus vidas. Comprender las experiencias de comunicación de los pacientes basadas en sus propias percepciones desempeña un papel crucial no solo en la toma de decisiones clínicas, sino también en la configuración de la filosofía general de la atención. En este contexto, las evaluaciones informadas por los pacientes sirven como un recurso poderoso para orientar y mejorar los servicios de salud^(9)^. Además, potenciar la participación del paciente contribuye a un intercambio de información más eficaz y conduce a una mejor calidad de la atención en general^(17)^. Estos hallazgos concuerdan con nuestros resultados y resaltan la influencia sustancial de las experiencias de comunicación tanto en la práctica clínica como en el enfoque más amplio de la atención.

Se han identificado cuatro enfoques distintos de las enfermeras hacia la comunicación centrada en el paciente en la atención al final de la vida: la oyente empática, la defensora orientada a la familia, la facilitadora que utiliza recursos y la cuidadora holística que invierte tiempo. Estas clasificaciones ilustran cómo los estilos de comunicación individuales de las enfermeras pueden moldear las experiencias de los pacientes. El enfoque del oyente empático, en particular, se alinea con la necesidad de sentirse comprendido, algo que los pacientes de nuestro estudio destacaron con frecuencia^(8)^.

Uno de los elementos más esenciales de la comunicación efectiva en los cuidados paliativos es garantizar que las personas que enfrentan un sufrimiento insuperable se sientan comprendidas. Un estudio cualitativo demostró el papel vital de la comunicación de apoyo y la empatía en este proceso. Los participantes informaron que no solo desarrollaron una mejor comprensión del sufrimiento ajeno, sino que también enfrentaron sus propios sentimientos de insuficiencia, convirtiéndose así en comunicadores más resilientes y eficaces. Tolerar los momentos de silencio, en particular, permitió a los pacientes expresar sus sentimientos y pensamientos con mayor libertad, abriendo la puerta a una sensación de paz interior tanto para el paciente como para el cuidador^(21)^. Estos hallazgos enfatizan que la comunicación en los cuidados paliativos debe basarse no solo en el intercambio de conocimientos técnicos, sino también en la resiliencia emocional y la comprensión mutua, lo cual concuerda estrechamente con los resultados del presente estudio.

Este estudio presenta varias limitaciones. Se llevó a cabo en un solo centro, lo que limita la generalización de los hallazgos a otras instituciones, regiones o sistemas de salud. Además, el diseño transversal impide establecer relaciones causales entre las variables y solo permite identificar asociaciones. El uso de datos autoinformados también pudo haber introducido sesgos relacionados con las condiciones emocionales o cognitivas de los participantes, incluido el sesgo de recuerdo. Los estudios futuros deberían incluir muestras multicéntricas y más diversas, utilizar diseños longitudinales e incorporar métodos cualitativos para proporcionar una comprensión más profunda de las experiencias de comunicación en los cuidados paliativos. A pesar de estas limitaciones, el estudio presenta varios puntos fuertes. Se utilizó la Escala de Barreras de Comunicación Centrada en el Paciente para evaluar las barreras de comunicación y se obtuvieron datos autoinformados directamente de los pacientes de cuidados paliativos. Este enfoque brinda una visión de primera mano sobre las experiencias de comunicación de los pacientes durante un período particularmente vulnerable y aporta una perspectiva centrada en el paciente que a menudo está subrepresentada en la investigación sobre cuidados paliativos.

Estos hallazgos tienen implicaciones importantes para la práctica clínica. Los profesionales de la salud que trabajan en entornos de cuidados paliativos deberían contar con el apoyo de programas estructurados de capacitación en comunicación que hagan hincapié en la empatía, la claridad y la participación activa del paciente. Abordar las necesidades no satisfechas de los pacientes en cuanto a atención e información puede ayudar a reducir las barreras de comunicación y mejorar la experiencia general de atención. Además, los cuidadores pueden desempeñar un papel de apoyo para facilitar la comunicación, ayudando a los pacientes a expresar sus inquietudes y a comprender mejor la información brindada. Sin embargo, es importante garantizar que la participación del cuidador no eclipse la propia voz del paciente en el proceso de comunicación. Fortalecer la participación del paciente en los procesos de intercambio de información y toma de decisiones puede mejorar aún más la comunicación centrada en el paciente en entornos de cuidados paliativos.

## CONCLUSIÓN

Este estudio identificó barreras clave en la comunicación centrada en el paciente entre las personas que reciben cuidados paliativos, en particular las necesidades insatisfechas de atención e información. Los resultados indican que la presencia de un cuidador se asocia con menores niveles de inseguridad en la comunicación con los profesionales de la salud, lo que sugiere un posible papel de apoyo de los cuidadores para aliviar las preocupaciones de los pacientes relacionadas con la comunicación. Sin embargo, la presencia del cuidador no se asoció significativamente con la percepción de prejuicios, lo que indica que esta dimensión de las barreras de comunicación puede verse influenciada por factores más allá de la mera participación del cuidador.

## Data Availability

El conjunto de datos que respaldan las conclusiones de este estudio está disponible previa solicitud al autor correspondiente.
